# Nox4 Overexpression as a Poor Prognostic Factor in Patients with Oral Tongue Squamous Cell Carcinoma Receiving Surgical Resection

**DOI:** 10.3390/jcm7120497

**Published:** 2018-12-01

**Authors:** Yen-Hao Chen, Chih-Yen Chien, Fu-Min Fang, Tai-Lin Huang, Yan-Ye Su, Sheng-Dean Luo, Chao-Cheng Huang, Wei-Che Lin, Shau-Hsuan Li

**Affiliations:** 1 Department of Hematology-Oncology, Kaohsiung Chang Gung Memorial Hospital and Chang Gung University College of Medicine, No.123, Dapi Rd., Niaosong Dist., Kaohsiung 833, Taiwan; alex8701125@gmail.com (Y.-H.C.); victor99@cgmh.org.tw (T.-L.H.); 2 Graduate Institute of Clinical Medical Sciences, College of Medicine, Chang Gung University, Taoyuan 333, Taiwan; 3 School of Medicine, Chung Shan Medical University, Taichung 402, Taiwan; 4 Department of Otolaryngology, Kaohsiung Chang Gung Memorial Hospital and Chang Gung University College of Medicine, No.123, Dapi Rd., Niaosong Dist., Kaohsiung 833, Taiwan; cychien3965@cgmh.org.tw (C.-Y.C.); yanyesu@cgmh.org.tw (Y.-Y.S.); rsd0323@cgmh.org.tw (S.-D.L.); 5 Department of Radiation Oncology, Kaohsiung Chang Gung Memorial Hospital and Chang Gung University College of Medicine, No.123, Dapi Rd., Niaosong Dist., Kaohsiung 833, Taiwan; fang2569@cgmh.org.tw; 6 Department of Pathology, Kaohsiung Chang Gung Memorial Hospital and Chang Gung University College of Medicine, No.123, Dapi Rd., Niaosong Dist., Kaohsiung 833, Taiwan; huangcc@cgmh.org.tw; 7 Biobank and Tissue Bank, Kaohsiung Chang Gung Memorial Hospital, No.123, Dapi Rd., Niaosong Dist., Kaohsiung 833, Taiwan; 8 Department of Diagnostic Radiology, Kaohsiung Chang Gung Memorial Hospital and Chang Gung University College of Medicine, No.123, Dapi Rd., Niaosong Dist., Kaohsiung 833, Taiwan; alex@cgmh.org.tw

**Keywords:** Nox4, oral tongue cancer, squamous cell carcinoma, surgery

## Abstract

Background: Nox4 has been reported to promote tumor progression of various types of cancer through many different pathways. The current study was designed to evaluate the prognostic significance of Nox4 in patients with oral tongue squamous cell carcinoma (OTSCC) receiving surgical resection. Methods: We retrospectively analyzed the 161 patients with OTSCC treated with surgical resection, including 81 patients with high expression of Nox4 and 80 patients with low expression of Nox4. Two OTSCC cell lines, SAS and SCC4, were used to investigate the proliferation activity. Results: The univariate and multivariable analyses showed that negative nodal metastasis and low expression of Nox4 were significantly associated with superior disease-free survival (DFS) and overall survival (OS). Western blotting analysis indicated that Nox4 was highly expressed in these two OTSCC cell lines and knockdown of Nox4 was successful by transfecting with Nox4 shRNA. In addition, these cell lines were also treated with a Nox4 inhibitor (GKT-137831) and the results showed GKT-137831 could inhibit the proliferation of OTSCC tumor cells in a dose-dependent manner. Conclusion: Our study suggests that Nox4 plays an important role in disease progression of OTSCC and Nox4 overexpression is a poor prognostic factor for patients with OTSCC who received surgical resection.

## 1. Introduction

Head and neck squamous cell carcinoma (HNSCC) is one of the leading cancers worldwide and there are an estimated 500,000 new cases being diagnosed annually [1]. In Taiwan, HNSCC is the sixth most common cancer and fifth leading cause of cancer-related deaths in men [2]. The tongue is the most frequent tumor location for intraoral cancers and tumors most often develop after a long history of tobacco use, alcohol or betel nut consumption and its incidence has increased in recent years. Treatment of oral tongue squamous cell carcinoma (OTSCC) includes a single surgical resection, radiotherapy, chemotherapy, targeted therapy or a combination of these modalities. Despite significant improvements in surgical techniques, chemotherapy, radiotherapy and targeted therapy in the last three decades, the outcome of patients with OTSCC still remains poor [3,4]. Recurrence is the most important prognostic factor and is primarily caused by aggressive local invasion and metastasis, leading to a poor prognosis and a negative quality of life. Thus, OTSCC remains a challenging disease to manage in the field of HNSCC. Therefore, identification of a reliable biomarker to correctly predict the likelihood of a recurrence to potentially reduce mortality in patients with OTSCC is an important research priority.

Nox4 is one of the nicotinamide adenine dinucleotide phosphate (NADPH) oxidases (NOXs) family and is the most frequently expressed isoform in these tumor cells [5]. It generates superoxide or hydrogen peroxide, produces reactive oxygen species (ROS) and been recognized as an important signal molecule in several cancers. Increased generation of ROS has been implicated in the pathogenesis of a variety of tumors [6,7], such as pancreatic cancer, breast cancer, non-small cell lung cancer and colon cancer [8,9,10,11]. Some previous studies have identified the biochemical links between Nox4 and cancer through several mechanisms, including angiogenesis, inflammatory cytokines, apoptosis resistance, histone modification, transforming growth factor-β and epidermal growth factor receptor pathway and so forth. [12,13,14,15,16,17]. Growing evidence confirms that there is a close correlation of Nox4 with cancer development and progression and the inhibition of Nox4 suppresses tumor growth and leads to cancer cell death [18].

However, the role of Nox4 in OTSCC remains unclear thus far. We postulate that Nox4 overexpression accounts for a novel mechanism that contributes to tumor progression and poor clinical outcome in patients with OTSCC. The aim of the present study was to elucidate the prognostic significance of Nox4 on survival in the progression of patients with OTSCC receiving surgical resection. Furthermore, in order to explore the function of Nox4 in cancer ell metabolism of OTSCC, we examined its expression in OTSCC cell lines and we evaluated the effect of Nox4 knockdown on cell proliferation in vitro.

## 2. Experimental Section

### 2.1. Patient Population

We retrospectively reviewed 1256 patients with OTSCC who were treated at Kaohsiung Chang Gung Memorial Hospital between January 2006 and December 2015. Among these 1256 patients with OTSCC, we first excluded those patients with a history of any second primary malignancy, distant metastasis, or who underwent preoperative chemotherapy or radiotherapy. After that, only those patients with OTSCC who received surgical resection as a curative treatment were included. Finally, a total of 161 patients with OTSCC with available paraffin blocks and medical records were identified. The tumor stage of each patient was determined according to the 7th American Joint Committee on Cancer (AJCC) staging system [19].

### 2.2. Immunohistochemistry

Immunohistochemistry staining was achieved using an immunoperoxidase technique and performed on slides (4 μm) of formalin-fixed paraffin-embedded tissue sections using primary antibodies against Nox4 (ab109225, 1:200, Abcam, Cambridge, MA, USA). Briefly, after deparaffinization and rehydration, slides were subjected to a heat-induced epitope retrieval in 10 mM citrate buffer (pH 6.0) in a hot water bath (95 °C) for 20 min. Immunodetection was performed using the LSAB2 kit (Dako, Carpinteria, CA, USA) followed by 3-3′-diaminobenzidine for color development and hematoxylin for counterstaining. For Nox4, incubation without the primary antibody was used as a negative control, while a slide of normal kidney tissue was used as a positive control. The staining assessment was independently carried out by two pathologists (S.L.W. and W.T.H.) without any information about clinicopathologic features or prognosis. Slides were examined at 200× and in each case, at least four sections were examined. We followed the previously published method to score the expression of Nox4 [18]. The percentage of Nox4 positive tumor cells for all neoplastic cells in the section was recorded. Nox4 overexpression was defined as the presence of staining in ≥50% of tumor cells.

### 2.3. Western Blot Analysis

For cell protein extraction, samples were homogenized in RIPA lysis buffer (50 mM Tris-HCl, pH 7.5, 150 mM NaCl, 1% NP-40, 0.5% Na-deoxycholate and 0.1% SDS). The protein concentration in each sample was estimated using a Bio-Rad Protein Assay (Bio-Rad, Hercules, CA, USA). Immunoblotting was performed according to standard procedures. Antibodies used in this study included polyclonal antibodies against Nox4 (ab109225, 1:200, Abcam, Cambridge, MA, USA) and β-actin (Sigma Aldrich, St Louis, MO, USA). The first antibodies were detected by incubation while secondary antibodies were conjugated to horseradish peroxidase (Bio/Can Scientific, Mississauga, ON, Canada) and developed using Western Lighting Reagent. The proteins were explored by X-ray films.

### 2.4. Cell Culture and Transfection

OTSCC cell lines SAS and SCC4 were established and purchased from Bioresource Collection and Research Center in Taiwan. These cell lines were cultured under standard conditions using Dulbecco′s modified Eagle′s medium (DMEM) with 10% fetal bovine serum, 1X MEM non-essential amino acids, 100 U/mL penicillin, 100 μg/mL streptomycin, 0.25 μg/mL Amphotericin B and 2.0 mmol/L L-glutamine.

To examine the cell proliferative activity of Nox4 in OTSCC, a 3-(4,5-dimethylthiazole-2-yl)-2,5-diphenyltetrazolium bromide (MTT) assay was performed on these cell lines. The procedure was performed as follows: each cell line (7000 cells) was incubated along with a control in a 96-well flat-bottomed plate in triplicate. After incubation for 96 h at 37 °C, 100 μL of MTT (3-(4,5-dimenthylthiazol-2-yl)-2,5-diphenyltetrazolium bromide, 0.5 mg/mL, Sigma, St. Louis, MO, USA) was added to each well and incubation was carried out for another four hours. Then, the supernatant was discarded and the crystal products were eluted with dimethyl sulfoxide (100 μL/well, Sigma). The colorimetric evaluation was tested using a spectrophotometer at 570 nm. The proliferation of each cell line harboring Nox4 overexpression was shown as a percentage of cell growth compared to the control cells.

To confirm the role of Nox4 in the malignant properties of OTSCC cells we transfected Nox4 shRNA into OTSCC cells to generate Nox4 knockdown cells. The above-mentioned OTSCC cell lines were used here. These cells were seeded in a 6 cm dish and allowed to grow to 50%–60% confluence for 24 h. Nox4 shRNA (40 nM) was purchased from RNAi Core of Academia Sinica (Taiwan) and then transfected into OTSCC cells using Hiperfect reagent according to the manufacturer′s protocol (Qiagen). To disrupt aggregates formed during lyophilization, shRNA was incubated at 90 °C for one minute and then at 37 °C for 60 min prior to the transfection procedure. The silencing efficiency was evaluated by western blotting 24 to 72 h after shRNA transfection. To examine the cell proliferative activity of Nox4 knockdown in OTSCC, an MTT assay was performed in these cell lines.

To test the role of Nox4 in the progression of OTSCC cells, we treated OTSCC cell lines, with or without a Nox4 inhibitor, with GKT-137831 (Selleck Chemicals, Houston, TX, USA), which is a novel and specific dual Nox1/Nox4 inhibitor. Each cell line (2500 cells) was incubated in 200 μL with GKT-137831 (0, 1, 5, 10, 20, 40, 80 and 100 μM) and control in a 96-well flat-bottomed plate in triplicate. To examine the cell proliferative activity of Nox4 inhibitor in OTSCC, an MTT assay was performed in these cell lines according to the above-mentioned procedures.

### 2.5. Statistical Analysis

For patient data, the statistical analyses were performed using the SPSS 19 software package (IBM, Armonk, NY, USA). Comparisons between the groups were performed using the chi-square test for categorical variable data. Disease-free survival (DFS) was computed from the time of surgery to the recurrence of cancer or death from any cause without evidence of recurrence. Overall survival (OS) was calculated from the date of diagnosis of the OTSCC to the date of death or last contact. The Kaplan–Meier method was used to estimate DFS and OS and the log-rank test was performed to evaluate the differences between the groups for univariate analysis. In a stepwise forward fashion, significant parameters at the univariate level were entered into a Cox regression model to analyze their relative prognostic importance. For cell line experiments, a *t*-test was used for the statistical analysis. Each experiment was carried out independently at least twice, with three repeats each. For all analyses, a *p*-value < 0.05 was considered statistically significant.

### 2.6. Ethics Statement

Study approval was obtained from the Chang Gung Medical Foundation Institutional Review Board (201700414B0) and all the patients provided the written informed consent. All the methods were carried out in accordance with the approved guidelines and the ethical standards of the World Medical Association Declaration of Helsinki.

## 3. Results

### 3.1. Patient Population

A total of 161 patients with OTSCC who received surgical resection were retrospectively examined at Kaohsiung Chang Gung Memorial Hospital. All of the 161 patients with OTSCC had an Eastern Cooperative Oncology Group performance status ≤1. The study group consisted of 148 male patients and 13 female patients with a median age of 53 years (range: 26 to 86 years). A total of 132 patients (82%) had a history of tobacco smoking and alcohol consumption was mentioned in 129 patients (80%). The tumor T status was found to be T1 in 46 patients (29%), T2 in 53 patients (33%), T3 in 13 patients (8%) and T4 in 49 patients (30%). Meanwhile, 93 patients (58%) were diagnosed as having N0 status, 22 patients (14%) as having N1 status, 44 patients (27%) as having N2 status and two patients (1%) as having N3 status. The tumor stage indicated that 36 patients (22%) had stage I, 34 patients (21%) had stage II, 23 patients (14%) had stage III, 62 patients (39%) had stage IVA and six patients (4%) had stage IVB. At the time of analysis, the median period of follow-up was 87.8 months for the 76 living survivors and 62.8 months (range: 2.3–117.6 months) for all 161 patients. The five-year DFS and OS rates were 68.9% and 47.2%, respectively. The clinicopathological parameters of these patients are shown in Table 1.

### 3.2. Silencing Nox4 Expression Reduces Tumor Cell Proliferation in Vitro

**Figure 1:**
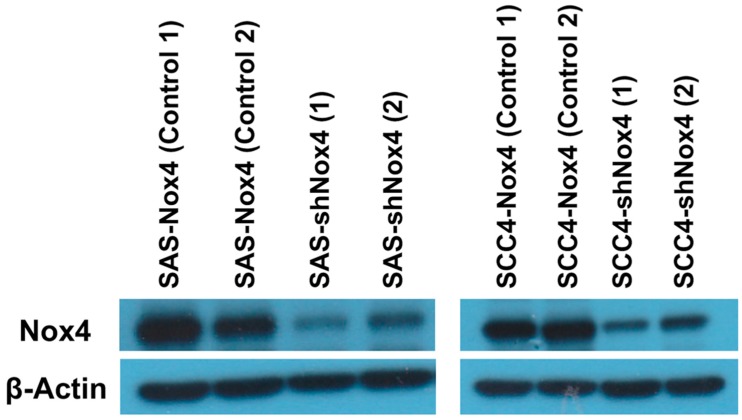
Western blotting analysis of Nox4 expression and knockdown of Nox4 in two oral tongue squamous cell carcinoma cell lines, SAS and SCC4.

**Figure 2:**
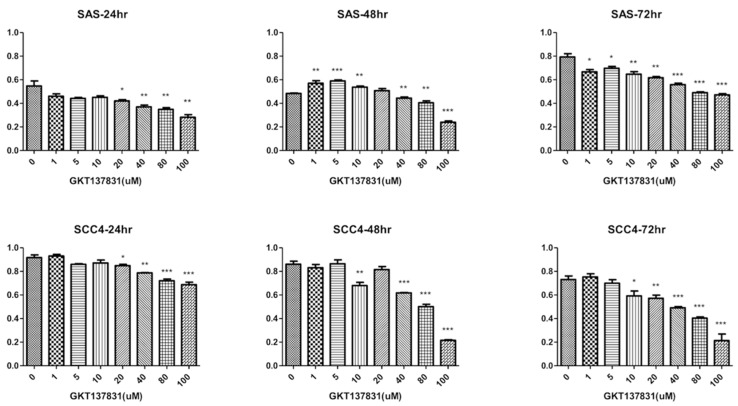
Nox4 inhibitor (GKT-137831) displays a growth inhibitory effect in a dose-dependent manner in the two oral tongue squamous cell carcinoma cell lines, SAS and SCC4. Columns, mean; bars, standard deviation. Significant difference in growth inhibition: * *p* < 0.05, ** *p* < 0.01 and *** *p* < 0.001.

In the present study, we performed western blotting analyses to determine Nox4 expression phenotype in OTSCC cell lines, SAS and SCC4. After that, these cell lines were stably transfected with Nox4 shRNA and knockdown of Nox4 was shown by western blotting (Figure 1). Furthermore, these cell lines were also treated with GKT-137831, a Nox4 inhibitor. These results showed GKT-137831 could inhibit the proliferation of tumor cells in a dose-dependent manner in SAS and SCC4 cell lines at 24th, 48th and 72nd hour after GKT-137831 treatment (Figure 2).

### 3.3. Expression of Nox4 and Clinical Outcome

**Figure 3:**
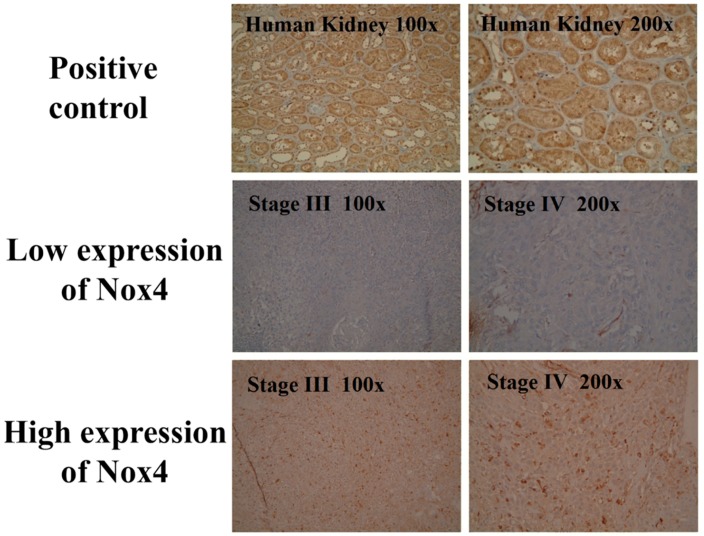
Immunohistochemical analysis of Nox4 expression in patients with oral tongue squamous cell carcinoma.

The expression of Nox4 in the immunohistochemical staining is shown in Figure 3. Among the 161 patients, 80 patients (49%) were classified as having a high expression of Nox4 and 81 patients (51%) had a low expression of Nox4. The baseline characteristics did not differ significantly between these two groups, including age, sex, cigarette smoking, alcohol consumption, tumor T status, tumor N status and tumor stage (Table 2).

**Figure 4:**
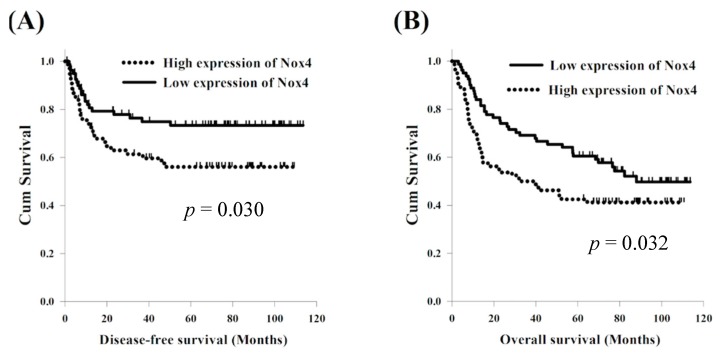
Comparison of survival curves for patients with oral tongue squamous cell carcinoma with high and low expression of Nox4. (**A**) Disease-free survival (**B**) Overall survival.

With respect to DFS, a univariate analysis found that sex and alcohol consumption were not statistically significant predictors of DFS. The 127 patients who were diagnosed at an age younger than 60 years were found to have superior DFS in comparison with the 34 patients diagnosed at an age older than 60 years (77.5 months versus 10.7 months, *p* < 0.001); meanwhile, 29 patients who never used cigarettes had superior DFS compared to the 132 smokers (not reach versus 40.2 months, *p* = 0.047). Significantly improved DFS was found in the 99 patients who had T1-2 status compared to the 62 patients who had T3-4 status (77.5 months versus 12.1 months, *p* = 0.004) and superior DFS was also found in the 93 patients without nodal metastasis compared to the other 68 patients with positive lymph node metastasis (not reach versus 13.4 months, *p* < 0.001). The 93 patients with stage I–III were found to have superior DFS in comparison with the 68 patients with stage IVA–IVB (*p* = 0.025). The 81 patients with a low expression of Nox4 had better DFS than the other 80 patients with a high expression of Nox4 (77.5 months versus 21.9 months, *p* = 0.047, Figure 4A). In a multivariable analysis, N0 status (*p* = 0.009, hazard ratio (HR): 0.47, 95% confidence interval (CI): 0.27–0.83) and a low expression of Nox4 (*p* = 0.0192, HR: 0.50, 95% CI: 0.28–0.89) were the independent prognostic parameters of better DFS.

With respect to OS, there were no statistically significant differences in parameters of sex and alcohol consumption in a univariate analysis. Significantly better OS was found in the 127 patients aged younger than 60 years than in the 34 patients aged older than 60 years (not reach versus 13.4 months, *p* = 0.001) and superior OS was also found in the 29 patients who never smoked in comparison to the 132 smokers (not reach versus 52.1 months, *p* = 0.026). The 99 patients with T1-2 were found to have better OS compared to the 62 patients with T3-4 (not reach versus 15.5 months, *p* = 0.001); meanwhile, 93 patients without nodal metastasis had superior OS than other 68 patients with positive nodal metastasis (not reach versus 15.5 months, *p* < 0.001). Better OS was found in the 93 patients who were classified as stage I–III compared to the 68 patients classified as having stage IVA–IVB (not reach versus 28.6 months, *p* < 0.001). The 81 patients with a low expression of Nox4 had better OS than the other 80 patients with a high expression of Nox4 (87.9 months versus 33.0 months, *p* = 0.032, Figure 4B). A multivariable analysis showed that T1-2 status (*p* = 0.019, HR: 0.59, 95% CI: 0.38–0.92), N0 (*p* = 0.001, HR: 0.47, 95% CI: 0.30–0.73) and a low expression of Nox4 (*p* = 0.011, HR: 0.57, 95% CI: 0.37–0.88) were the independent prognostic parameters of superior OS. These univariate and multivariable survival analyses are shown in Table 3.

## 4. Discussion

Nox4 has been found to promote tumor progression of many types of cancer through various pathways. Nox4 may induce cancer cell progression through promoting tumor angiogenesis. In Nox4 knockout mice with fibrosarcoma, vessel density analysis showed a significant reduction in tumor vascularization, leading to the attenuation of hypoxia-inducible factor 1-alpha, vascular endothelial growth factor, glucose transporter 1 and adrenomedullin [15]. Inflammatory cytokines are one of the critical mediators in inflammation-associated cancer, especially interleukin-6 (IL-6). In non-small cell lung cancer, Nox4 expression is positively correlated with IL-6 expression and exogenous IL-6 treatment significantly enhances Nox4 signaling [16]. The same result was also described in renal cell carcinoma cells [13]. Another possible mechanism for Nox4 inducing cancer progression includes histone modification, transforming growth factor-β and epidermal growth factor receptor (EGFR) pathway and so forth. [12,14,17].

The current study found that Nox4 and lymph node metastasis were both prognostic factors for patients with OTSCC in the univariate and multivariable analyses. Several studies have confirmed that the presence of neck lymph node metastasis is the most reliable prognostic factor of regional or distant treatment failure in patients with OTSCC [20,21,22,23]. Recently, growing evidence confirmed the role of Nox4 in the disease progression of various cancer types. Zhang et al. showed that Nox4 was upregulated and promoted tumor cell proliferation in vitro and in vivo and overexpression of Nox4 was closely correlated to tumor stage and contributed to the poor prognosis of patients with non-small cell lung cancer [18]. Lin et al. also demonstrated that Nox4 promoted tumor cell proliferation and apoptosis, migration and invasion and Nox4 overexpression was highly correlated with tumor invasion depth, positive lymph node numbers, distant metastasis and poor prognosis of patients with colorectal cancer [24]. Nox4 also plays a crucial role in regulating gastric cancer cell growth. In a Chinese study, Nox4 expression was correlated with tumor size and poor prognosis in 90 patients with gastric cancer and knockdown of NOX4 expression blocked cell proliferation and the expression of Cyclin D1, BAX and so on in vitro. Nox4 promoted cell proliferation via activation of the GLI1 pathway and overexpression of GLI1 reversed the suppression of tumor cell growth induced by silencing NOX4. Furthermore, overexpression of Nox4 enhanced expression of GLI1 and knockdown of GLI1 expression reduced the effects induced by Nox4 overexpression [25]. A Japanese study, reported by Ito et al., also demonstrated that Nox4 was highly expressed in several oral squamous cell carcinoma cell lines and NOXs knockdown markedly suppressed cell viability and induced apoptosis; in addition, NOXs suppression significantly enhanced the cisplatin-induced cytotoxic effect [26].

In head and neck cancer, an EGFR inhibitor is one of the major therapeutic modalities for HNSCC and is routinely used in clinical practice. Autophagy has been reported to be one of the possible mechanisms of resistance to chemotherapy or EGFR inhibitors and Nox4 plays a critical role in mediating this effect [27]. Chronic inflammation has been confirmed to be strongly associated with tumor invasion, migration and metastasis through increased secretion of pro-inflammatory cytokines, such as IL-2, IL-6, tumor necrosis factor-α and so forth. The antitumor activity of EGFR inhibitors is suppressed by activation of Nox4-mediated pro-inflammatory pathways and knockdown of Nox4 reduced EGFR inhibitor-induced pro-inflammatory cytokine expression [14]. Several studies have highlighted the contribution of the microenvironment to tumor progression and cancer-associated fibroblasts (CAFs) are related to poor prognosis in various cancer types, including head and neck cancer [28,29,30]. Upregulation of Nox4 expression was strongly correlated with myofibroblastic-CAFs, contributing to decreased cancer-specific survival rates. Suppression of Nox4 was found to revert the myofibroblastic-CAF phenotype, prevent myofibroblastic-CAF accumulation and slower tumor proliferation [31].

The 8th edition of the AJCC staging manual has been introduced into clinical practice in 2018 [32]. There are most significant changes to oral cavity cancer staging, including changes to the T and N staging categories, depth of invasion and extranodal extension (ENE). Several studies have been designed to investigate the importance of the 8th edition of the AJCC staging system. Mascitti et al. showed that the 8th edition of the AJCC criteria is more suitable for better stratification of patients with OTSCC and the implementation of ENE and lymph node ratio to pathological N classification are indicated to identify patients with poor prognosis [33]. Another study reported by Pollaers et al. revealed that the 8th edition of the AJCC staging system supports better DFS discrimination between overall stages and between T categories in patients with oral cavity squamous cell carcinoma [34]. In our study, although the tumor stage of each OTSCC patient who underwent surgical resection was determined according to the 7th AJCC staging system, the results showed negative nodal metastasis and low expression of Nox4 were significantly associated with superior DFS and OS and the N0 status was not changed whether in the 7th or 8th AJCC staging system.

This study had several limitations. First, it was a retrospective analysis at a single institution with a relatively small sample size. Second, we did not explore the comprehensive mechanisms of Nox4 and downstream pathways, nor investigate how Nox4 overexpression promotes tumor cell proliferation, invasion and metastasis.

However, to the best of our knowledge, this study, at present, covers the largest series of patients with OTSCC who received surgical resection and may thus be useful for understanding the role of Nox4 in the prognosis of OTSCC.

## 5. Conclusions

The results of our study suggest that Nox4 plays an important role in disease progression of OTSCC and Nox4 overexpression is a poor prognostic factor of patients with patients with OTSCC who received surgical resection. Additional studies are warranted in order to clarify the complex mechanism of Nox4 and downstream pathways in patients with OTSCC.

## Figures and Tables

**Table 1:** Clinicopathological parameters in 161 patients with oral tongue squamous cell carcinoma receiving surgical resection.

Characteristics	
Age	53 years old (26–86)
Sex	
Male	148 (92%)
Female	13 (8%)
Cigarette smoking	
Absent	29 (18%)
Present	132 (82%)
Alcohol consumption	
Absent	32 (20%)
Present	129 (80%)
T status	
1	46 (29%)
2	53 (33%)
3	13 (8%)
4	49 (30%)
N status	
0	93 (58%)
1	22 (14%)
2	44 (27%)
3	2 (1%)
Tumor stage	
I	36 (22%)
II	34 (21%)
III	23 (14%)
IVA	62 (39%)
IVB	6 (4%)
Nox4 expression	
High	80 (51%)
Low	81 (49%)

**Table 2:** Comparison of clinicopathological parameters in 161 patients with oral tongue squamous cell carcinoma receiving surgical resection.

Characteristics			
	High expression of Nox4 (*N* = 80)	Low expression of Nox4 (*N* = 81)	*p*-Value
Age	53 years old (32–86)	51 years old (26–70)	
Sex			
Male	75 (94%)	73 (90%)	0.40
Female	5 (6%)	8 (10%)	
Cigarette smoking			
Absent	14 (17%)	15 (18%)	0.87
Present	66 (83%)	66 (82%)	
Alcohol consumption			
Absent	16 (20%)	16 (20%)	0.97
Present	64 (80%)	65 (80%)	
T status			
1–2	47 (59%)	52 (64%)	0.48
3–4	33 (41%)	29 (36%)	
N status			
0	46 (58%)	47 (58%)	0.95
1–3	34 (42%)	34 (42%)	
Tumor stage			
I–III	35 (44%)	35 (43%)	0.95
IVA–IVB	45 (56%)	46 (57%)	

**Table 3:** Univariate and multivariable analysis of disease-free survival and overall survival in in 161 patients with oral tongue squamous cell carcinoma receiving surgical resection.

Characteristics	No. of Patients	Univariate Analysis		Multivariate Analysis		Univariate Analysis		Multivariate Analysis	
		Median DFS (months)	*p*-Value	HR (95% CI)	*p*-Value	Median OS (months)	*p*-Value	HR (95% CI)	*p*-Value
Age									
<60 years	127 (79%)	77.5	<0.001 *			NR	0.001 *		
≥60 years	34 (21%)	10.7				13.4			
Sex									
Male	393 (97%)	46.0	0.24			57.7	0.14		
Female	11 (3%)	NR				NR			
Cigarette smoking									
Absent	29 (18%)	NR	0.047 *			NR	0.026 *		
Present	132 (82%)	40.2				52.1			
Alcohol consumption									
Absent	32 (20%)	57.4	0.55			NR	0.42		
Present	129 (80%)	41.2				64.5			
T status									
1 + 2	99 (62%)	77.5	0.004 *			NR	0.001 *	0.59 (0.38–0.92)	0.019 *
3 + 4	62 (38%)	12.1				15.5			
N status									
0	93 (58%)	NR	<0.001 *	0.47 (0.27–0.83)	0.009 *	NR	<0.001 *	0.47 (0.30–0.73)	0.001 *
1 + 2 + 3	68 (42%)	13.4				15.5			
Tumor stage									
I–III	93 (58%)	NR	0.025 *			NR	<0.001 *		
IVA–IVB	68 (42%)	NR				28.6			
Nox4 expression									
High	80 (49%)	21.9	0.030 *			33.0	0.032 *		
Low	81 (51%)	77.5		0.50 (0.28–0.89)	0.018 *	87.9		0.57 (0.37–0.88)	0.011 *

DFS: disease-free survival; OS: overall survival; NR: not reached; HR: hazard ratio; CI: confidence interval * Statistically significant.

## Abbreviations

HNSCC	Head and neck squamous cell carcinoma
OTSCC	Oral tongue squamous cell carcinoma
NADPH	Nicotinamide adenine dinucleotide phosphate
NOXs	Nicotinamide adenine dinucleotide phosphate oxidases
ROS	Reactive oxygen species
DMEM	Dulbecco′s modified Eagle′s medium
DFS	Disease-free survival
OS	Overall survival
IL-6	Interleukin-6
EGFR	Epidermal growth factor receptor
CAFs	Cancer-associated fibroblasts
ENE	Extranodal extension
